# MLL4 mediates differentiation and tumor suppression through ferroptosis

**DOI:** 10.1126/sciadv.abj9141

**Published:** 2021-12-10

**Authors:** Shaun Egolf, Jonathan Zou, Amy Anderson, Cory L. Simpson, Yann Aubert, Stephen Prouty, Kai Ge, John T. Seykora, Brian C. Capell

**Affiliations:** 1 Department of Dermatology, University of Pennsylvania Perelman School of Medicine Philadelphia, PA 19104, USA.; 2 Penn Epigenetics Institute, University of Pennsylvania Perelman School of Medicine, Philadelphia, PA 19104, USA.; 3 National Institutes of Diabetes and Digestive and Kidney Diseases, National Institutes of Health, Bethesda, MD 20892, USA.; 4 Abramson Cancer Center, University of Pennsylvania Perelman School of Medicine, Philadelphia, PA 19104, USA.; 5 Department of Genetics, University of Pennsylvania Perelman School of Medicine, Philadelphia, PA 19104, USA.; 6 Penn Institute for Regenerative Medicine, University of Pennsylvania Perelman School of Medicine, Philadelphia, PA 19104, USA.

## Abstract

The epigenetic regulator, *MLL4* (*KMT2D*), has been described as an essential gene in both humans and mice. In addition, it is one of the most commonly mutated genes in all of cancer biology. Here, we identify a critical role for Mll4 in the promotion of epidermal differentiation and ferroptosis, a key mechanism of tumor suppression. Mice lacking epidermal *Mll4*, but not the related enzyme *Mll3* (*Kmt2c*), display features of impaired differentiation and human precancerous neoplasms, all of which progress with age. Mll4 deficiency profoundly alters epidermal gene expression and uniquely rewires the expression of key genes and markers of ferroptosis (*Alox12*, *Alox12b*, and *Aloxe3*). Beyond revealing a new mechanistic basis for *Mll4*-mediated tumor suppression, our data uncover a potentially much broader and general role for ferroptosis in the process of differentiation and skin homeostasis.

## Teaser

This study reveals how epigenetic errors drive skin cancer initiation via altered cell fate and impaired ferroptosis.

## INTRODUCTION

Epigenetic dysregulation is a “hallmark” of human cancer (*1*), with almost half of all human cancers bearing mutations in epigenetic regulators. Squamous cell carcinomas (SCCs) occur on a variety of epithelial surface tissues and harbor the highest rates of mutations in chromatin-modifying enzymes among cancers (*2*). The epigenetic regulator MLL4 (KMT2D) is considered an essential gene in both human and mice (*3*, *4*) and is one of the most frequently mutated genes across all of human cancer (*5*–*9*). Notably, mutations in *MLL4* have been associated with both early clone formation in normal epithelial tissues (*10*–*12*), as well as with aggressive and metastatic forms of cutaneous SCC (cSCC) (*13*–*15*), the second most common of all human malignancies (*16*). The emerging picture, from both these and other studies (*5*, *6*), suggests that *MLL4* typically accumulates loss of function mutations that impair its ability to suppress both the initiation and progression of cancer. Despite this, while previous work from our laboratory has identified a critical role for MLL4 in epidermal gene regulation (*17*), the mechanisms behind MLL4-mediated tumor suppression in the skin is virtually unknown. Given the pervasive nature of keratinocyte cancers (i.e., cSCC and basal cell carcinoma, which collectively outnumber all other human cancers) (*16*), as well as *MLL4* mutations in this tissue, understanding the underlying mechanisms at play offers the potential for identifying previously unknown therapeutic targets.

## RESULTS

### Mll4 deficiency in the epidermis promotes altered differentiation and neoplastic proliferation

**Fig. 1.**
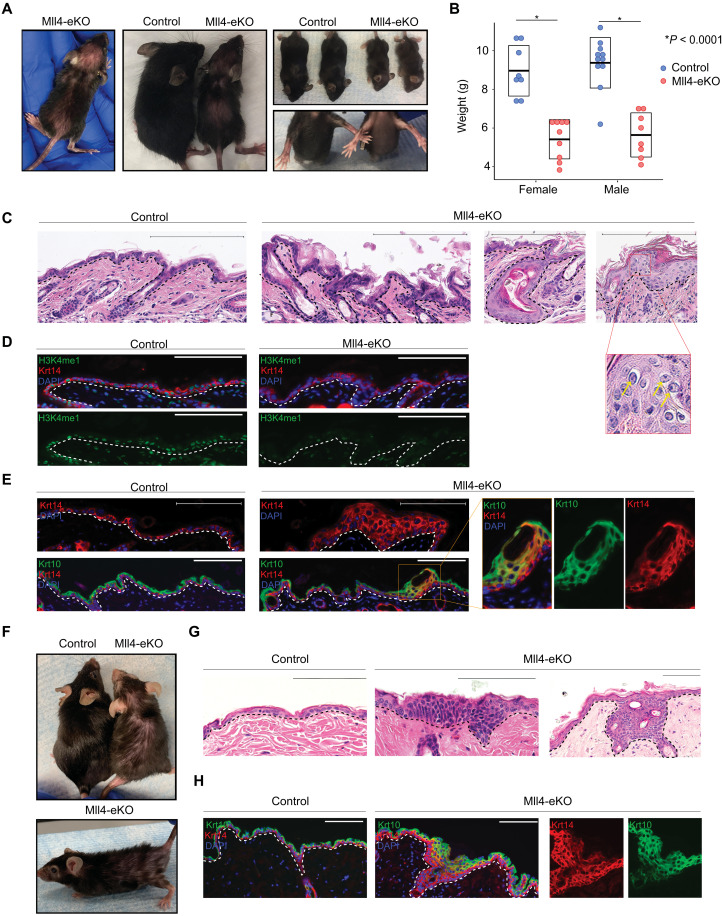
Mll4 deficiency in the epidermis promotes altered differentiation and neoplastic proliferation. (**A**) Representative 3-week-old Mll4-eKO and control mice. (**B**) Weights of either male or female 3-week-old Mll4-eKO (red) and control (blue) mice (*n* = 17 to 19 mice per genotype, all *P* < 0.0001). (**C**) H&E staining of 3-week-old Mll4-eKO and control mice epidermis (*n* = 17 to 19 mice per genotype). Scale bars, 200 μM. (**D**) IF staining of 3-week-old Mll4-eKO and control mice epidermis for H3K4me1 (green), Krt14 (red), and DAPI (blue) (*n* = 5 mice per genotype). (**E**) IF staining of 3-week-old Mll4-eKO and control mice epidermis for Krt14 (red), Krt10 (green), and DAPI (blue) (*n* = 5 mice per genotype; box denotes zoomed-in section). (**F**) Representative 1- to 2-year-old Mll4-eKO and control mice. (**G**) H&E histological staining of 1- to 2-year-old Mll4-eKO and control mice epidermis (*n* = 3 mice per genotype). (**H**) IF staining of 1- to 2-year-old Mll4-eKO and control mice epidermis for Krt14 (red), Krt10 (green), and DAPI (blue) (*n* = 3 mice per genotype). Black and white dashed lines delineate the epidermal and dermal boundary. Scale bars, 100 μM, unless otherwise noted.

To begin to address this knowledge gap, we created mice with epidermal-specific deletions of *Mll4*, as the total body knockout of *Mll4* is embryonic lethal at day 9.5 (*4*). We used a previously described conditional knockout of *Mll4* in which the catalytic SET domain is flanked by two floxP sites (exons 50 and 51) and whereby Cre-mediated deletion of the floxed catalytic SET domain destabilizes the protein and results in tissue-specific deletion of Mll4 (*18*). We crossed this genetic model with keratin 14 Cre (Krt14-Cre)–expressing mice to generate mice with epidermal deletions of *Mll4* (Krt14-Cre; *Mll4*^fl/fl^ or “Mll4-eKO”) (fig. S1A). Mll4-eKO mice were born in the expected Mendelian ratios (fig. S1B) with no visible differences from littermate controls during the first week of life. However, at approximately 21 days after birth, Mll4-eKO mice displayed a notable cutaneous phenotype marked by visibly red, scaly skin with scattered regions of substantial hair thinning (Fig. 1A). In addition to this, Mll4-eKO mice were visibly smaller (Fig. 1A) and weighed 40% less than controls (fig. S1C). This weight difference was consistent across genders (Fig. 1B). At the histological level, Mll4-eKO mice displayed multiple consistent abnormalities in the epidermis as revealed by hematoxylin and eosin (H&E) staining, including overall tissue disorganization and scattered regions of notable epidermal hyperplasia and hyperkeratosis (Fig. 1C). These regions of hyperplasia and hyperkeratosis were notable for both the presence of atypical keratinocytes with large nuclei and increased numbers of mitotic cells (Fig. 1C, inset), all features commonly observed in the precancerous form of cSCC in humans known as actinic keratoses. As Krt14 is also expressed in the oral and esophageal epithelia, we also examined these tissues. Similar to the epidermis, we observed scattered, albeit more rare regions of, epithelial hyperplasia in the oral epithelium of the tongue (fig. S1D), while the esophageal epithelium appeared largely unremarkable (fig. S1E).

In the skin, Mll4-eKO mice also presented with sporadic improperly formed hair follicles that resulted in the formation of cyst-like structures (Fig. 1C), consistent with the observed hair thinning and regions of alopecia seen in these mice (Fig. 1A). Given Mll4’s role in catalyzing histone H3 lysine 4 monomethylation (H3K4me1), we examined the global levels of this modification that marks gene enhancers by immunofluorescence (IF) and observed significantly reduced levels (Fig. 1D). These features were not observed in littermate controls, which had a histologically normal epidermis.

Given these histological anomalies, we next assessed the various layers of the epidermis that correspond with progressive differentiation states. As expected, control samples had a single layer of Krt14-positive staining cells marking the proliferative basal stem cell layer and a distinct, more terminally differentiated Krt10-staining spinous layer (Fig. 1E). In contrast, Mll4-eKO mice displayed a multilayered, expanded Krt14 layer suggestive of a potential failure of upward differentiation of the basal stem cells (Fig. 1E). Further, the expanded Krt14 layers in the epidermis of Mll4-eKO mice commonly contained dual Krt14- and Krt10-positive staining keratinocytes further suggesting a state of dysfunctional epidermal differentiation (Fig. 1E). As increased inflammation from barrier disruption can drive epidermal hyperplasia in different contexts (*19*), we investigated levels of inflammation using both H&E and CD3 T cell staining. In both cases, Mll4-eKO did not demonstrate any difference in the amount or numbers of immune cells (Fig. 1, F and G), suggesting that the significant hyperplasia we observed was not just secondary to increased inflammation.

To observe how Mll4 loss affects epidermal homeostasis and differentiation over time, we aged Mll4-eKO mice for at least 1 year and some for up to 2 years. The epidermal phenotype persisted in Mll4-eKO and appeared to worsen with age, including progression of both the grossly visible hair loss and scaly, hyperkeratotic skin (Fig. 1F). Similarly, at the histological level, the abnormal epidermal phenotype seemed to progress over time, as Mll4-eKO mice displayed regions of neoplastic proliferation, many of which appeared to be more infiltrative and consistent with early cSCC lesions in humans (Fig. 1G). Consistent with this observation, an expanded Krt14/Krt10 dual-staining layer was also observed in these mice (Fig. 1H). Together, these data demonstrate that Mll4 loss significantly impairs proper epidermal homeostasis and differentiation in vivo.

### Loss of Mll4 leads to a profoundly altered transcriptome characterized by a loss of key genes involved in differentiation and lipid metabolism

**Fig. 2.**
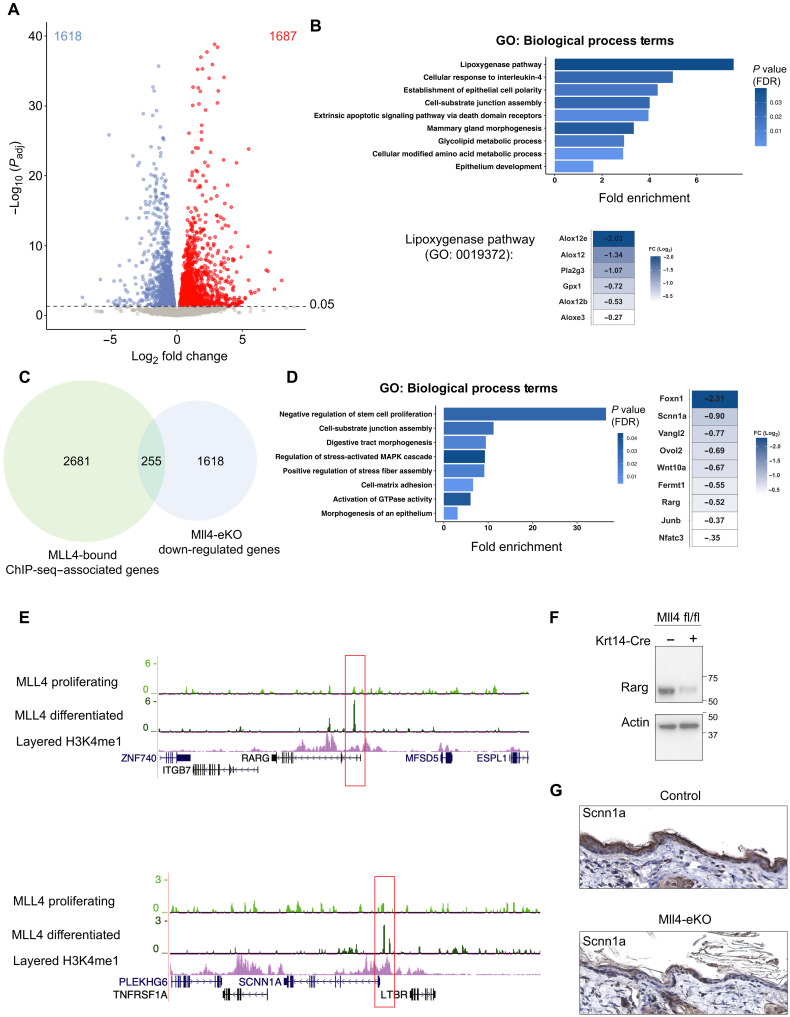
Loss of Mll4 leads to a profoundly altered transcriptome characterized by a loss of key genes involved in differentiation and lipid metabolism. (**A**) Differentially expressed genes (1618 down-regulated genes and 1687 up-regulated genes) in isolated bulk epidermis of 3-week-old Mll4-eKO and control mice. Dashed line denotes adjusted *P* value (*P*_adj_) of 0.05 (*n* = 3 to 4 mice per genotype). (**B**) GO biological process analysis of Mll4-eKO differentially expressed down-regulated genes (1618 genes). Down-regulated genes driving the top GO term “lipoxygenase pathway” are highlighted. (**C**) Intersection of all MLL4-bound region-associated genes in NHEKs and down-regulated genes in Mll4-eKO mice (255 genes). (**D**) Left: GO biological process analysis of the intersection in (D) (255 genes). Right: List of key epidermal differentiation genes enriched in GO terms from (D) intersection. (**E**) ChIP-seq for MLL4 in proliferating or differentiated primary human keratinocytes at key epidermal differentiation (*RARG*) and barrier (*SCNN1A*) genes listed in Fig 2E (*n* = 2 to 3 per condition). (**F**) Immunoblot of Rarg and actin isolated from bulk epidermis of 3-week-old Mll4-eKO and control mice. Molecular ladder shown to the right of blot (*n* = 2 mice per genotype). (**G**) IHC staining of Sccn1a of 3-week-old Mll4-eKO and control mice epidermis (*n* = 3 mice per genotype. Scale bar, 200 μM). FDR, false discovery rate; FC, fold change.

To begin to understand the mechanisms behind these changes, we performed RNA sequencing (RNA-seq) on the epidermis from littermate control and Mll4-eKO mice. As expected, given Mll4’s critical role in transcriptional regulation, Mll4-eKO mice demonstrated substantial transcriptional alterations with roughly equal number of significantly down-regulated and up-regulated genes (Fig. 2A and table S1). Gene Ontology (GO) analysis of down-regulated genes demonstrated a significant enrichment of genes involved in lipoxygenase activity and lipid metabolism (Fig. 2B). This was particularly compelling given the essential role of both of these processes in normal epidermal homeostasis and barrier formation (*20*, *21*). Underscoring the critical nature of many of these down-regulated genes, mutations in a number of them are causative in a variety of human genetic skin disorders such as *Col7a1*, *Fermt1*, *Slurp1*, *Alox12b*, and *Aloxe3* (*22*–*24*).

In contrast, genes up-regulated in the Mll4-eKO mice were enriched for numerous genes and GO terms involved in various aspects of fatty acid metabolism, such as fatty acid synthesis, β-oxidation, and elongation (fig. S2A and table S1). This was intriguing given that fatty acid metabolism has been implicated in promoting both metastasis and treatment resistance in cancer (*25*–*28*). Notably, many of these significantly up-regulated genes have been reported to be involved in driving cancer invasion and metastasis such as *Acat* (*Soat1*), *Cd36*, *Fabp5*, *Mgll*, *Scd1*, *Slc1a5*, and *Slc7a11* (*29*, *30*). For example, CD36 has been shown to mark metastasis-initiating cells in oral SCC and targeting it inhibits metastasis in vivo (*31*).

To examine whether Mll4 might directly regulate these genes, we differentiated primary normal human epidermal keratinocytes (NHEKs) in vitro and mapped Mll4 binding by chromatin immunoprecipitation sequencing (ChIP-seq). Intersection of Mll4 peaks with genes significantly down-regulated in Mll4-eKO mice demonstrated substantial overlap (Fig. 2C). These again included numerous genes involved in epidermal development, differentiation, and barrier formation such as the retinoic acid receptor gene, *RARG*, and *SCNN1A* (Fig. 2, D and E) (*32*, *33*). Notably, the ChIP-seq data demonstrated that the MLL4 peaks emerged at many of these genes only in the differentiated NHEKs and were not present in the proliferating, undifferentiated NHEKs (Fig. 2E). Specifically, there were 1389 peaks that were unique to the differentiated state, while only 209 were unique to the proliferating stem cell state (Fig. 2, B and C, and table S2). Most of the peaks (1870) were common to both (Fig. 2, B and C, and table S2). Consistent with previous data (*17*), motif analysis demonstrated that the top transcription factor motifs for MLL4 binding enrichment were those for the master epithelial transcription factor, p63, and p53 (fig. S2D). Western blotting and immunohistochemistry (IHC) confirmed that these gene expression changes resulted in reduced protein levels for retinoic acid receptor gamma (RARγ) and sodium channel epithelial 1 subunit alpha (SCNN1A), respectively (Fig. 2, F and G). Collectively, these data demonstrate that mice lacking Mll4 in the epidermis display broad gene expression alterations marked by the loss of key genes and pathways involved in epidermal differentiation and barrier formation and consistent with the significant phenotype observed in Mll4-eKO mice. Furthermore, MLL4 binds directly at enhancers near many of these genes during the course of differentiation, indicating a potentially direct role in activating their expression.

### In contrast to Mll4 loss, Mll3 deficiency leads to modest effects in the epidermis

**Fig. 3.**
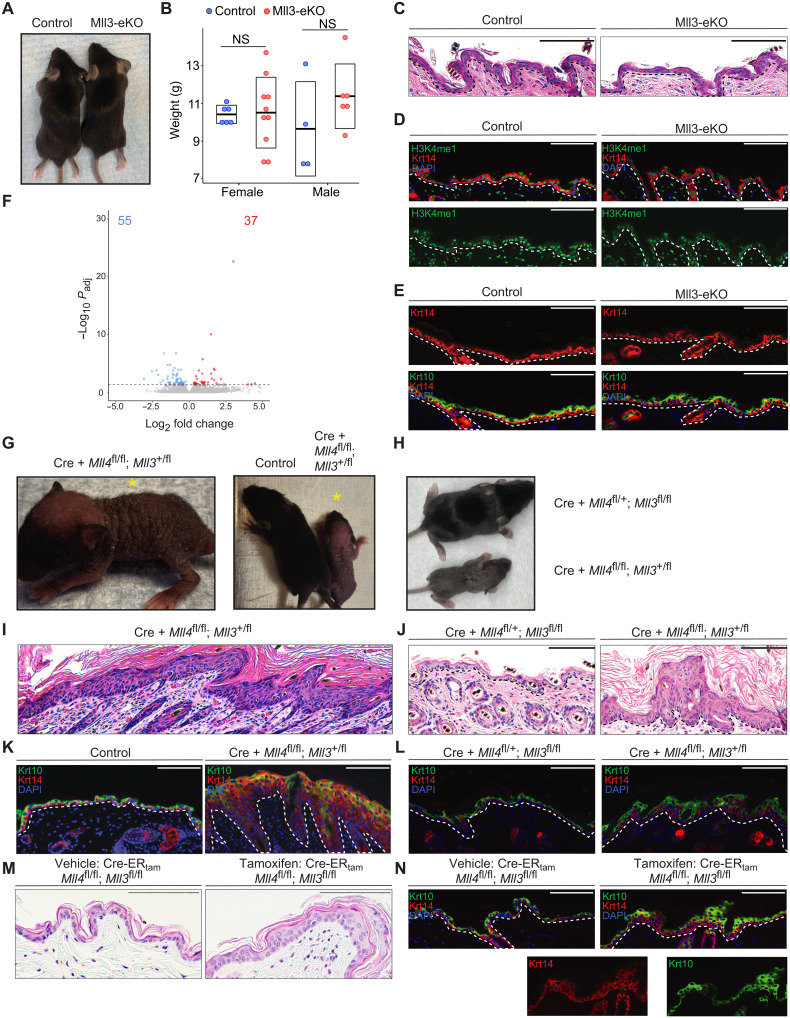
In contrast to Mll4 loss, Mll3 deficiency leads to modest effects in the epidermis. (**A**) Representative 3-week-old Mll3-eKO and control mice. (**B**) Weights of either male or female 3-week-old Mll3-eKO (red) and control (blue) mice (*n* = 9 to 16 mice per genotype, *P* = 0.49). NS, not significant. (**C** to **E**) Comparison of 3-week-old Mll3-eKO and control mice epidermis by (C) H&E staining, (D) IF staining of H3K4me1 (green), Krt14 (red), and DAPI (blue) or (E) IF staining of Krt14 (red), Krt10 (green), and DAPI (blue) (*n* = 3 mice per genotype). (**F**) Differentially expressed genes (55 down-regulated genes and 37 up-regulated genes) in isolated bulk epidermis of Mll3-eKO and control mice. Dashed line denotes adjusted *P* value of 0.05 (*n* = 6 mice per genotype). (**G**) Representative 1-week-old Krt14-Cre(+); *Mll4*^fl/fl^; *Mll3*^+/fl^ mouse alone (yellow asterisk) (left) or beside a littermate control (right). (**H**) Littermate sibling Krt14-Cre(+); *Mll4*^fl/+^; *Mll3*^fl/fl^ (top) and Krt14-Cre; *Mll4*^fl/fl^; *Mll3*^+/fl^ (bottom) mice compared. (**I**) H&E staining of a Krt14-Cre(+); *Mll4*^fl/fl^; *Mll3*^+/fl^ mouse epidermis (*n* = 3 mice per genotype). (**J**) Littermate sibling Krt14-Cre(+); *Mll4*^fl/+^; *Mll3*^fl/fl^ and Krt14-Cre(+); *Mll4*^fl/fl^; *Mll3*^+/fl^ mice compared by (J) H&E histological staining of the epidermis, and (**K**) IF staining for Krt14 (red), Krt10 (green), and DAPI (blue) (*n* = 3 mice per genotype). (**M** and **N**) Comparison of the epidermis of vehicle and tamoxifen-treated Krt14-CreER^tam^; *Mll4*^fl/fl^; *Mll3*^fl/fl^ mice by (M) H&;E staining or (N) IF staining of Krt14 (red), Krt10 (green), and DAPI (blue) (*n* = 2 mice per group, one vehicle control and one genotype control). Black and white dashed lines delineate the epidermal and dermal boundary. Scale bars, 100 μM, unless otherwise noted.

Similar to *MLL4*, the related histone methyltransferase, *MLL3* (*KMT2C*), also catalyzes H3K4me1and is frequently mutated in numerous human cancers, including SCCs (*5*–*9*). Therefore, we also wanted to investigate the role of MLL3 in epidermal biology and tumor suppression. To do this, we took a similar approach to generate mice with epidermal-specific deletions of *Mll3* by crossing Krt14-Cre mice with mice carrying *Mll3* alleles with loxP sites flanking a 61–amino acid region of the catalytic SET domain. Cre-mediated recombination results in deletion of the targeted region (fig. S3A) and results in destabilization and loss of the full protein (*34*, *35*). In clear contrast to Mll4-eKO mice, mice lacking Mll3 in the epidermis (“Mll3-eKO”) did not display any obvious phenotypic alterations beyond subtle changes around the eye (Fig. 3A and fig. S3B). There was no significant change in weight between control and Mll3-eKO mice (Fig. 3B). Histologically, the Mll3-eKO mice also did not demonstrate significant or consistent changes in epidermal thickness or organization (Fig. 3C), and H3K4me1 levels were only minimally reduced (Fig. 3D). Consistent with these findings, Krt14 and Krt10 staining did not suggest alterations in differentiation dynamics in contrast to Mll4-eKO mice (Fig. 3E). Last, RNA-seq of Mll3-eKO mice showed that a loss of *Mll3* in the epidermis only led to modest alterations in gene expression (Fig. 3F and table S3), with minimal overlap with those genes altered by Mll4 deficiency (fig. S3C). Together, these data demonstrated that Mll4 plays a more critical role in epidermal gene regulation, homeostasis, and differentiation than Mll3.

To determine whether Mll3 may compensate on some level for the loss of Mll4 in the epidermis or whether it was completely dispensable even in the setting of Mll4 deficiency, we attempted to generate mice lacking both Mll3 and Mll4 in the epidermis. Here, we observed that mice lacking Mll4 and one copy of Mll3 were even more severely affected than mice lacking just Mll4, suggesting that Mll3 does perform some compensatory functions for Mll4 in its absence. These Krt14-Cre(+); *Mll4*^fl/fl^; *Mll3*^+/fl^ mice displayed markedly wrinkled, scaly skin with sparse hair (Fig. 3G). Notably, these mice were significantly more affected than mice lacking all Mll3 and one copy of Mll4 (Krt14-Cre(+); *Mll4*^fl/+^; *Mll3*^fl/fl^) (Fig. 3H). At the histological level, Krt14-Cre(+); *Mll4*^fl/fl^; *Mll3*^+/fl^ mice presented with an even more thickened epidermis and expanded epidermis than mice lacking only Mll4 (Mll4-eKO) (Fig. 3I and fig. S3D). In contrast, Krt14-Cre(+); *Mll4*^fl/+^; *Mll3*^fl/fl^ mice (fig. S3, E to G) appeared less affected than both Krt14-Cre(+); *Mll4*^fl/fl^; *Mll3*^+/fl^ mice and Mll4-eKO mice (Fig. 3, J to L). We found that we were unable to generate any live mice lacking both Mll3 and Mll4, suggesting that at least one copy of these two related enzymes is required for epidermal development (fig. S3H). To test this further, we created a tamoxifen-inducible line of these mice using a Krt14-CreER^tam^ system. Using this approach, we demonstrated that deletion of both *Mll3* and *Mll4* in the adult epidermis recapitulated several of the phenotypic manifestations of embryonic loss of Mll4 and/or Mll3 (Fig. 3, M and N). In contrast to the constitutive model, these data demonstrated that complete deletion of both enzymes in the adult epidermis was compatible with life. Collectively, these observations supported the conclusions that Mll4 is indeed the more critical H3K4 monomethylase in the epidermis and that at least one copy of *Mll3* or *Mll4* is required for epidermal development and viability.

### Mll4 deficiency impairs ferroptosis via rewired gene expression

**Fig. 4.**
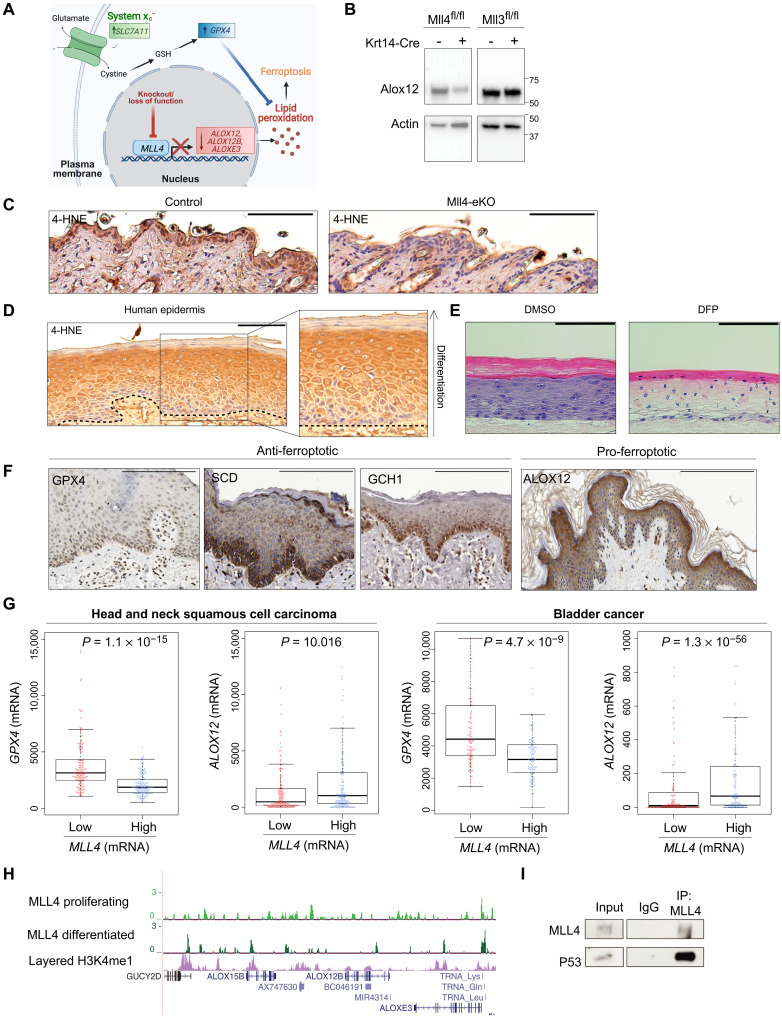
Mll4 deficiency impairs ferroptosis via rewired gene expression. (**A**) Schematic demonstrating that knockout of *Mll4* results in lost expression of pro-ferroptosis genes (*Alox12*, *Alox12b*, and *Aloxe3*) and up-regulation of key anti-ferroptosis genes (*Slc7a11* and *Gpx4*) ultimately decreasing cellular lipid peroxides and inhibiting ferroptosis. (**B**) Immunoblot of Alox12 and actin isolated from bulk epidermis of 3-week-old Mll4-eKO, Mll3-eKO, and control mice. Molecular ladder shown to the right of blot (*n* = 2 mice per genotype). (**C**) IHC staining of 4-HNE in 3- to 8-week-old Mll4-eKO and control mice epidermis (*n* = 5 mice per genotype). (**D**) IHC staining of 4-HNE in human epidermis (*n* = 4 patient samples). Dashed line indicates epidermal and dermal boundary. (**E**) 3D human skin organoids treated with 1 mM DFP display marked alterations in epidermal differentiation including a complete loss of the granular layer and parakeratosis in comparison to DMSO-treated controls. (**F**) IHC staining of anti-ferroptosis (GPX4, SCD, and GCH1) or pro-ferroptosis (ALOX12) proteins in human epidermis obtained from the Human Protein Atlas. Scale bars, 200 μM. (**G**) Comparison of GPX4 and ALOX12 expression in the lowest one-fourth and highest one-fourth of MLL4-expressing tumors in 515 human head and neck SCCs (left) or 411 human bladder cancers (right) obtained from TCGA. All *P* < 0.05. (**H**) ChIP-seq for MLL4 in proliferating or calcium differentiated human keratinocytes at the Alox family genes (*n* = 2 to 3 per condition). (**I**) Co-IP of MLL4, followed by immunoblotting of p53 in human keratinocytes (*n* = 3). Scale bar, 100 μM, unless otherwise noted. IgG, immunoglobulin G.

To begin to explain the more severe phenotype observed with Mll4 loss in comparison to Mll3, we took advantage of the substantially different transcriptional profiles that these mice displayed and the fact that the changes appeared to reflect the observed unique phenotypic manifestations. In particular, the most notable difference observed in the Mll4-eKO mice was the significant loss of expression of key genes involved in lipoxygenase activity such as *Alox12*, *Alox12b*, and *Aloxe3* (Fig. 2D). While lipoxygenases are well established as key enzymes during the process of epidermal differentiation (*21*), we were particularly intrigued by their emerging role in the relatively recently discovered form of programmed tumor suppressive cell death known as ferroptosis that is characterized by lipid peroxidation (Fig. 4A) (*36*–*38*). For example, Arachidonate 12-Lipoxygenase, 12S Type (Alox12) is required for p53-mediated tumor suppression via ferroptosis (*36*). Many human cancers are noted to have a loss of one copy of *ALOX12* on chromosome 17p (*39*, *40*), and variants in *ALOX12* have also been associated with human SCCs (*41*). In addition to the loss of these key lipoxygenase enzymes, two of the key genes that suppress ferroptosis through the elimination of lipid peroxides, *Slc7a11* and *Gpx4* (*36*–*38*, *42*), were concomitantly significantly up-regulated in the Mll4-eKO mice (Fig. 4A). Furthermore, among the up-regulated fatty acid metabolism genes we identified in the Mll4-eKO mice (Fig. 2A), the gene stearoyl coenzyme A (CoA) desaturase-1 (*Scd1*), the rate-limiting enzyme monounsaturated fatty acid synthesis, has been shown to both promote cancerous invasion and protect against ferroptosis (*43*, *44*).

Given these changes, we first verified Alox12 loss at the protein level in Mll4-eKO, but not Mll3-eKO mice (Fig. 4B). We then used a marker of lipid peroxidation and ferroptosis, 4-hydroxynoneal (4-HNE) (*37*, *45*), to assess the impact that loss of Mll4 might have. Notably, we observed a marked reduction in 4-HNE staining in Mll4-eKO mice (Fig. 4C) that was not observed in Mll3-eKO mice, suggesting that a potentially significant reduction in Alox-mediated ferroptosis might occur in the setting of Mll4 deficiency. More speculatively, these data provoked the hypothesis that ferroptosis may actually play a critical role in the promotion of epidermal differentiation. The precise mechanism by which keratinocytes enucleate and undergo programmed cell death during terminal differentiation is poorly understood. Therefore, we stained human skin samples to assess how the ferroptosis marker, 4-HNE, may appear. We observed a clear progressive increase in staining intensity as cells progressed toward terminal differentiation, with the highest levels of staining observed in the highly metabolically active stratum granulosum (Fig. 4D), precisely where keratinocytes make the transition to becoming enucleated and forming the stratum corneum where they ultimately slough off the skin. Given the iron-dependent nature of both ferroptosis and the lipoxygenase enzymes such as Alox12, we next tested the effects of depleting iron on epidermal differentiation and stratification by treating engineered three-dimensional (3D) human skin organoids (*46*) with the iron chelator, deferiprone (DFP). DFP treatment markedly disrupted normal epidermal differentiation and stratification throughout the epidermis (Fig. 4E). This was characterized by notable clearing of cytoplasm in the spinous layers, a complete loss of granular layers and keratohyalin granules, and parakeratosis (i.e., retained nuclei in the stratum corneum) (Fig. 4E). Collectively, these results suggest that ferroptosis may play a critical role in the promotion of normal epidermal differentiation and that loss of Mll4 function impairs this process to result in features of precancerous neoplasms in the epidermis.

In support of these observations, data from the Human Protein Atlas (*47*) confirmed the enrichment of anti-ferroptotic regulators like GPX4, SCD1, and GCH1 in the proliferative basal stem cell layer, while pro-ferroptotic proteins like Alox12 were enriched in the stratum granulosum where the highest levels of 4-HNE were observed (Fig. 4F). In addition, other publicly available data from The Cancer Genome Atlas (TCGA) (*7*, *8*) showed that lower expression of MLL4 was significantly associated with both lower expression of ALOX12 and higher expression of GPX4 in human head and neck SCC as well as bladder cancer (Fig. 4G). In support of a direct role for MLL4 in promoting the expression of these lipoxygenase genes, ChIP-seq demonstrated that MLL4 binds at potential regulatory elements and enhancers adjacent to these Alox-family genes (Fig. 4H). Furthermore, in addition to our previous data showing that MLL4 interacts with the transcription factor, p63 (*17*, *48*), we identified that MLL4 also interacts with p53 (Fig. 4I). Both p53 and p63 have been shown to induce the expression of Alox12 to promote both epidermal differentiation (*49*) and tumor suppression (*36*, *50*), respectively. This is also consistent with our ChIP-seq motif analysis showing that the main transcription factor binding motifs of MLL4 are p63/p53 sites (fig. S2D). Together, these data support a model whereby Mll4 is critical for the activation of key lipoxygenase genes such as *Alox12* to promote both epidermal differentiation and barrier formation and, in turn, tumor suppression via ferroptosis (Fig. 4A). Beyond this direct regulation of lipoxygenase genes, the aberrant up-regulation of ferroptosis-suppressing genes such as *Gpx4*, *Slc7a11*, and *Scd1* in Mll4-eKO mice supports a potentially lipogenic and ferroptosis-resistant state that confers a cellular advantage to promote the neoplasms observed in these mice.

## DISCUSSION

In summary, our results offer unique insight into the primary importance of MLL4 as compared to MLL3 in the skin epidermis. As *MLL4* (*KMT2D*) loss of function mutations are among the most common events in human cancer, the results presented here offer a novel mechanism by which these mutations may promote both the initiation and progression of cancer, particularly in the setting of keratinocyte cancers, which collectively outnumber all other human malignancies (*16*). Given the ability to pharmacologically induce ferroptosis through inhibitors of targets such as GPX4 or SLC7A11 (*37*, *38*), these data suggest a potentially new therapeutic strategy for treating epithelial cancers like SCC, particularly in the setting of *MLL4* mutations. In addition, in the context of recent evidence demonstrating that drugs that induce ferroptosis can also operate synergistically with immunotherapies (*37*, *51*), there may exist even more potential uses when used in combination. More generally, as these results implicate ferroptosis in the general process of epidermal differentiation and skin homeostasis, it suggests that future studies may uncover a role for dysregulated ferroptosis not only in skin cancers but also potentially in a variety of diverse skin disorders as well.

## MATERIALS AND METHODS

### NHEK isolation and culture

Primary epidermal progenitors were isolated from deidentified discarded neonatal human foreskin obtained by Core B of the Penn Skin Biology and Diseases and Resource-based Center. Primary human keratinocyte cultures used for organotypic epidermis were isolated from human foreskin procured by the Penn Skin Biology and Disease Resource-based Center under a protocol (#808224) approved by the University of Pennsylvania Institutional Review Board and determined to be exempt for formal informed consent for use of discarded, deidentified tissues. Foreskin was incubated at 4°C for 12 hours in dispase II (2.4 U/ml). Sterile forceps were used to separate the underlying dermis. The epidermal sheet was transferred to a 60-mm tissue culture plate, incubated in 0.25% trypsin for 10 min at 37°C, and then neutralized with 1 ml of fetal bovine serum (FBS). Sterile forceps were used to scrape the epidermal sheet against the dish to dissociate cells. The suspension was passed through a 40-μm strainer and then centrifuged at 200*g* for 5 min. The cell pellet was resuspended in 5 ml of keratinocyte medium (described below). Epidermal progenitors were cultured in a 50:50 mix of 1× keratinocyte serum-free medium (SFM) supplemented with human recombinant epidermal growth factor (EGF) and bovine pituitary extract combined with medium 154 supplemented with human keratinocyte growth supplement and 1% penicillin-streptomycin (10,000 U/ml) at 37°C.

### Murine models

All animal protocols were reviewed and approved by the Institutional Animal Care and Use Committee of the University of Pennsylvania. Mice were maintained on a mixed C57BL/6 background on a standard light-dark cycle. Mice carrying Mll4SET floxed alleles, Mll3SET floxed alleles, or a combination of both of these were crossed with Krt14-Cre transgenic mice. Krt14-Cre: Mll4SET^fl/fl^ (Mll4-eKO), Krt14-Cre: Mll3SET^fl/fl^ (Mll3-eKO), triple floxed combinations (Krt14-Cre: Mll4SET^fl/fl^ Mll3SET^+/fl^ or Krt14-Cre: Mll4SET^+/fl^ Mll3SET^fl/fl^), and the quadruple flox combination (Krt14-Cre: Mll4SET^fl/fl^ Mll3SET^fl/fl^) were considered mutants. Unless noted otherwise, littermates homozygous for the Mll alleles of interest lacking Krt14-Cre were used as controls. A similar approach was used using Krt14-CreER^tam^ (005107, Jackson Laboratory) transgenic mice. To activate inducible knockouts, tamoxifen (T5648-1G, Sigma-Aldrich) was resuspended in corn oil (C8267, Sigma-Aldrich) and used for injections. Both genotype controls and corn oil (vehicle) controls were used to account for the potential effects of tamoxifen. Generation of Mll4SET^fl/fl^ and Mll3SET^fl/fl^ mice has been described elsewhere (*18*, *34*). For genotyping, polymerase chain reaction (PCR) was done using a Thermo Phire Animal Tissue PCR kit (F140WH) using the following primers: Mll3 (forward: GTCATCGGTGTGGTCTGAATGA and reverse: AACCGGAAGGAGAAGCTTTATGA), Mll4 (forward: CAGTTGAGCTAGTCAAGTGATT and reverse: TTCAATGTGGAGGGGAGTGACAG), and Cre (forward: GAACCTGATGGACATGG and reverse: AGTGCGTTCGAACGCTAGAGCCTGT). Unless otherwise stated, all experimental mice were a mix of male and female. The number of animals used per experiment is stated in the figure legends.

### Murine RNA and protein extraction

Murine epidermis was dissociated from the dermis before isolation of bulk RNA and protein. Time points were selected as to not include the anagen phase of the hair cycle, which compromises the dissociation process. Following euthanasia of 21-day-old mice, the skin was dissected, and the underlying fat pad was removed using a scalpel. The resulting tissue was floated dermis side down in dispase (5 U/ml; Corning) in PBS for 40 min at 37°C. The epidermis was then removed using a scalpel, and for RNA, the epidermis was flash-frozen in TRIzol and stored at −80°C until RNA isolation. RNA was extracted using an RNeasy kit (#74104, QIAGEN) at the same time and date for all mice belonging to a single experimental cohort (i.e., RNA-seq) regardless of the date of murine euthanasia to reduce batch effects and stored at −80°C. For protein, epidermis was placed directly into cold PBS and centrifuged at 4°C for 5 min at 2500 rpm. To the resulting pellet, protein lysis buffer (Cell Signaling Technology) containing a protease inhibitor cocktail was added, and the mixture was homogenized, sonicated, rotated at 4°C for 10 min, and then centrifuged at 4°C at full speed for 10 min. Lysates were quantified using the Bradford assay. Frozen lysates were stored at −80°C.

### Real-time PCR

Complementary DNA was obtained using a high-capacity RNA-to-DNA kit (#4368814, Thermo Fisher Scientific). For quantitative real-time PCR, Power SYBR Green PCR Master Mix (#4367659, Thermo Fisher Scientific) was used. Primer sequences are available on request. Quantitative real-time PCR data analysis was performed by first obtaining the normalized cycle threshold (CT) values (normalized to glyceraldehyde-3-phosphate dehydrogenase and 18*S* ribosomal RNA), and the 2 − ΔΔ*C*t method was applied to calculate the relative gene expression. ViiA 7 Real-Time PCR System was used to perform the reaction (Applied Biosystems). The average and SDs were assessed for significance using a Student’s *t* test. All *P* are noted in the figure legends and were considered significant if *P* < 0.05 and nonsignificant (NS) if *P* > 0.05.

### RNA sequencing

RNA-seq libraries were prepared at the same time for all samples belonging to a single experimental cohort to reduce batch effects. All RNA-seq libraries were prepared using the NEBNext Poly(A) mRNA magnetic isolation module, followed by NEBNext Ultra Directional RNA library preparation kit for Illumina. Library quality was checked by Agilent BioAnalyzer 2100, and libraries were quantified using the Library Quant Kit for Illumina. Libraries were then sequenced using a NextSeq500 platform [75–base pair (bp) single-end reads]. All RNA-seq was aligned using RNA STAR (*52*) under default settings to *Mus musculus* GRCm38 fragments per kilobase per million mapped fragments, and generation and differential expression analysis were performed using DESeq2 (*53*). Statistical significance was obtained using an adjusted *P* value (*P*_adj_) generated by DESeq2 of less than 0.05.

### ChIP sequencing

ChIP-seq was performed as described previously [(*54*) and (*17*)]. Briefly, keratinocytes cultured in 10-cm^2^ dishes were fixed in 1% formaldehyde for 5 min, and fixation was quenched with the addition of glycine to 125 mM for an additional 5 min. Cells were harvested by scraping from plates and washed twice in 1× PBS before storage at −80°C. ChIP extracts were sonicated for 15 min in a Covaris sonicator. All ChIPs were performed using 500 μg of extract and 2 μg of antibody per sample (anti-MLL4 ). Thirty microliters of Protein G Dynabeads was used per ChIP. ChIP DNA was also used to make sequencing libraries using a NEBNext Ultra DNA library preparation kit for Illumina. Library quality was checked by Agilent BioAnalyzer 2100, and libraries were quantified using the Library Quant Kit for Illumina. Libraries were then sequenced using a NextSeq500 33 platform (75-bp, single-end reads). After sequencing, all data were demultiplexed from the raw reads using Illumina’s BCL2FASTQ from BaseSpace. Further ChIP-seq analysis is described below.

### ChIP-seq data processing

ChIP-seq data were analyzed as previously described (*55*). Briefly, FASTQ reads from lanes 1 to 4 were combined for each sample and aligned to *Homo sapiens* genome (hg19 UCSC) using bowtie2 (version 2.1) (*56*), allowing one mismatch per seed (-N 1) and reporting one alignment per read (-k 1). Alignment files were filtered with SAMtools (version 1.1) (*57*) to remove unmapped reads (-F 4) and reads with a mapping quality inferior to 10 (-q 10). After sorting and indexing, alignment files were further filtered to remove reads mapped to mitochondrial chromosome or unplaced contigs (chrM, chrUn, and chrN_random). MLL4 (KMT2D) peaks were called with HOMER (version 4.6) (*58*). Tag directories were generated for each replicate using an estimated fragment length of 150 bp, allowing one tag per base pair (makeTagDirectory -fragLength 150 -tbp 1). Tag directories from replicates were combined with HOMER makeTagDirectory using an estimated fragment length of 150 bp (-fragLength 150). MLL4 peaks were called for each condition (proliferating or differentiated NHEK) from merged tag directories using their corresponding input with HOMER findPeaks (-style factor -size 200 -fragLength auto -F 2 -fdr 0.005), yielding 967 MLL4 peaks in proliferating NHEKs and 2954 MLL4 peaks in differentiated NHEKs. MLL4 peaks from both conditions were combined, sorted, merged, and filtered to remove peaks with less than 1 read per million, yielding 3468 peaks, and condition-selective MLL4 peaks were defined by a fold-change threshold of 2, yielding 1870 common peaks, 209 proliferation-selective MLL4 peaks, and 1369 differentiation-selective MLL4 peaks. Analysis of transcription factor–binding motifs at each peak set was performed using HOMER findMotifsGenome.pl script with default parameters, scaling sequence logos by information content (−bits).

### GO analyses

All GO analyses were performed using PANTHER at http://pantherdb.org/ to determine statistically overrepresented GO terms using Fisher’s exact test under the category “biological process.” *P* values for GO terms are false discovery rate statistics. The top eight plotted GO terms represent the GO terms with the highest fold enrichment under PANTHER’s default hierarchical clustering categorization. GO term figures are generated using ggplot2.

### Co-immunoprecipitation experiments

Co-immunoprecipitation (Co-IP) experiments were performed as previously described (*52*). Briefly, 30 ml of magnetic Protein G Dynabeads were washed twice in 1 ml of 0.5% bovine serum albumin (BSA), resuspended in 250 ml of 0.5% BSA, and conjugated for 1 to 2 hours at 4°C under rotation with 1 mg of antibodies against either MLL4 or immunoglobulin G (IgG) as a negative control. About 500,000 proliferating epidermal progenitors cells were harvested from a 10-cm culture plate at 50% confluence and lysed for 1 hour at 4°C under rotation in 250 ml of IP buffer [20 mM tris (pH 7.5), 134 mM NaCl, 1 mM CaCl_2_, 1% NP-40, and 10% glycerol] supplemented with freshly made 1 mM MgCl_2_, 1:100 Halt protease and phosphatase inhibitor cocktail (catalog no. 78440, Thermo Fisher Scientific), and benzonase at 12.5 U/ml. Benzonase is critical for the efficient release of chromatin-bound proteins to the supernatant, and MgCl_2_ is critical for its activity. Cell lysates were centrifuged at top speed for 10 min. Cell lysate (500 mg) was incubated overnight at 4°C with antibody-conjugated magnetic beads previously washed three times with 1 ml of 0.5% BSA. Immunoprecipitates were collected using a magnet; washed four to five times with IP buffer devoid of MgCl_2_, protease/phosphatase inhibitors, and benzonase; then boiled with NuPage loading dye, and analyzed by Western blotting. Antibodies used during Co-IP and subsequent immunoblotting include anti-MLL4 (Sigma-Aldrich, HPA035977-100UL), anti-P53 (EMD-Millipore, MABE327), and IgG (Santa Cruz, sc-2005).

### Immunoblotting

Samples were separated by electrophoresis in 4 to 20% SDS–polyacrylamide gel electrophoresis gels with 20 mg per lane, transferred to a polyvinylidene difluoride membrane, and blotted with antibodies. Secondary horseradish peroxidase–conjugated secondary antibodies (Santa Cruz Biotechnology) and Amersham ECL Prime Western Blotting Detection Reagents (catalog no. RPN2232, GE Healthcare) were used for detection. Antibodies used during immunoblotting include anti-actin (Santa Cruz, sc-1616), anti-RARγ (Santa Cruz, sc-7387), and anti-Alox12 (Santa Cruz, sc-365194).

### Histology

Mouse dorsal and ventral skin tissues were processed for histological examination by Core A of the Penn Skin Biology and Disease Resource-based Center and mounted on frost-free slides. H&E staining was processed by Penn Skin Biology and Disease Resource-based Center Core A. A Leica DM6 B microscope was used to observe and capture representative images. Exposure times and microscope intensity were kept constant for all human samples and across mouse littermate comparisons.

### Immunohistochemistry

Tissue slides were baked for 1 hour at 65°C, deparaffinized in xylene, and rehydrated through a series of graded alcohols. After diH_2_O washes, slides were treated with antigen unmasking solution (1:100; SKU H-3300-250, Vector Laboratories) at 95°C for 10 min according to the manufacturer’s protocol. Blocking and primary antibody binding were detected using a VECTASTAIN Elite ABC-HRP system according to the manufacturer’s instructions (catalog no. PK-6200, Vector Laboratories). After O/N primary antibody (see antibody table) incubation at 4°C, endogenous peroxidases were blocked with 3% hydrogen peroxide in MeOH for 10 min at room temperature (RT), treated with biotinylated secondary anti-mouse/rabbit antibody at RT for 90 min, and treated with ABC reagent (VECTASTAIN Elite ABC-HRP Kit, PK-6200, Vector Laboratories) for 30 min at RT. The staining was visualized with 3,3′-diaminobenzidine (catalog no. SK-4100, Vector Laboratories) as peroxidase substrate. Exposure times were synchronized so that all tissues samples within an antibody group were exposed to 3, 3’-diaminobenzidine (DAB) for the exact same time. All slides were counterstained with hematoxylin (Hematoxylin QS, H-3404, Vector Laboratories) for 35 s at RT, dehydrated in ethanol, cleared in xylene, and mounted with VectaMount (permanent mounting medium, H-5000, Vector Laboratories). Antibodies used for IHC include anti-4HNE (abcam, ab46545), anti-SCNN1a (Proteintech, 10924-2-AP), and anti-ALOX12 (Santa Cruz, sc-365194).

### Immunofluorescence

Mouse tissues slides were deparaffinized with xylene, rehydrated with alcohol, and treated with Targeting Unmasking Fluid (1:3; catalog no. Z000R.0000, Pan Path) at 90°C for 10 min according to the manufacturer’s protocol. Slides were permeabilized with 0.5% Triton X-100 in Dulbecco’s PBS for 10 min and incubated in BlockAid Blocking Solution (Thermo Fisher Scientific, catalog no. B10710) for 1 hour at RT in a humidity chamber and incubated O/N in primary antibody. Following secondary antibody treatment (see antibody table) for 30 min at RT, the sections were mounted with ProLong Gold with 4′,6-diamidino-2-phenylindole (DAPI) (catalog no. P36935, Thermo Fisher Scientific). Antibodies used for IF include anti-KRT14 (abcam, ab7800), anti-KRT10 (abcam, ab7638), anti-H3K4me1 (abcam, ab8895), anti-RARγ (Santa Cruz, sc-7387), and anti-CD3 (Biolegend, 300324).

### Weight collection

Following euthanasia, mice at 21 days of age were measured for total body weight. Data figures are the composite of multiple litters of Mll4-eKO or littermate controls. A one-way analysis of variance (ANOVA), followed by Tukey’s multiple comparisons test was used to calculate significant differences between groups for all mice when stratified by gender and genotype (Fig. 1B). A Student’s *t* test was used to calculate significance between groups when considered for genotype only (fig S1C). All *P* are noted in figure legends and were considered significant if *P* < 0.05 and nonsignificant (NS) if *P* > 0.05.

### 3D human skin organoids

The 3D human skin organoids were performed as previously described (*46*). Briefly, J2 3T3 fibroblasts were grown in Dulbecco’s modified Eagle’s medium (DMEM) + 10% FBS. Cells were released from culture plates using 0.25% trypsin for 5 min at 37°C, resuspended in DMEM + 10% FBS, and counted using a hemocytometer to determine the volume needed to obtain 0.75 million to 1 million fibroblasts per organotypic culture. The required volume was centrifuged in a 50-ml sterile conical tube at 200*g* for 5 min, and the supernatant was removed. The fibroblast cell pellet was resuspended in 1/10 the final required volume (2 ml per culture) of 10× collagen resuspension buffer (1.1 g of NaHCO_3_ plus 2.39 g of Hepes in 50 ml of 0.05 N NaOH) and held on ice. One-tenth the final volume of 10× DMEM (Sigma-Aldrich) was then added, and the cells were mixed by vigorous pipetting. Purified high-concentration rat tail collagen I (Corning) was added and diluted with sterile diH_2_O to a final concentration of 4 mg/ml of the final volume. NaOH (0.05 N) was added to a pH of approximately 7. The collagen-fibroblast slurry was mixed by inverting, and then, 2 ml was pipetted into the top chamber of a six-well transwell insert (Corning) placed within a deep-well six-well tissue culture plate (Corning). The fibroblast-collagen matrices were allowed to polymerize at 37°C for 60 min. Then, the matrices were submerged in DMEM + 10% FBS and placed at 37°C overnight. The next day, NHEKs were trypsinized, resuspended in DMEM + 10% FBS, counted to collect 1 million cells per culture, and centrifuged at 200*g* for 5 min, and the supernatant was discarded. The NHEK pellet was resuspended in E-medium supplemented with EGF (5 ng/ml; Sigma-Aldrich) to a volume of 2 ml per culture. The DMEM was removed from both the top and bottom chambers of the transwell plates containing the collagen-fibroblast matrices. A total of 2 ml of NHEKs (1 million cells) were seeded atop each matrix in the top transwell chamber, and 14 ml of E-medium with EGF (5 ng/ml) was added to the bottom chamber. The cultures were placed at 37°C overnight. The next day, the medium was aspirated from both the top and bottom chambers of the transwell. To place the NHEK monolayers at an air-liquid interface and induce stratification, 10 ml of E-medium (without EGF supplementation) was added only to the bottom chamber of the transwell, and the cultures were grown at 37°C for up to 12 days, feeding 10 ml of E-medium every other day. DFP (#379409, Sigma-Aldrich) was used at 1 mM, and cultures were treated on day 6 at the air-liquid interface with either DFP or dimethyl sulfoxide (DMSO) vehicle, then allowed to grow for 72 more hours, and fixed in formalin for routine histology (*N* = 5 unique NHEK isolates were used and showed similar effects).

### TCGA analysis

RNA-seq datasets from head and neck SCC and bladder cancer were obtained from TCGA (https://tcga-data.nci.nih.gov/tcga). Original RNA expression values (normalized read counts) were used for downstream analyses. For each cancer type, samples were ranked by MLL4 expression levels and were evenly divided into four groups. Comparisons were performed between the group of samples with the highest MLL4 expression and the group of samples with lowest MLL4 expression. One-sided Wilcoxon rank-sum tests were used to compute significance.

### Human protein atlas

IHC data derived from the Human Protein Atlas can be found at www.proteinatlas.org/

### Statistical analyses

All statistical analyses were performed using R or GraphPad Prism 8. Details of each statistical test are included in Materials and Methods. Sample sizes and *P* values are included in the figure legends or main figures. Investigators were not blinded during experiments or outcome assessment.

## Notes

View/request a protocol for this paper from *Bio-protocol*.
