# Supplementary material for: MLL4 mediates differentiation and tumor suppression through ferroptosis
Source: Sci Adv. 2021 Dec 10;7(50):eabj9141. doi: 10.1126/sciadv.abj9141 (PMC8664260; doi:10.1126/sciadv.abj9141)
Supplement: Supplementary file 1 — Figs. S1 to S3 Legends for data files S1 to S3 [file sciadv.abj9141_sm.pdf]

Science Advances
AAAS

Supplementary Materials for

MLL4 mediates differentiation and tumor suppression through ferroptosis

Shaun Egolf, Jonathan Zou, Amy Anderson, Cory L. Simpson, Yann Aubert, Stephen Prouty, Kai Ge, John T. Seykora, Brian C. Capell*

*Corresponding author. Email: capellb@pennmedicine.upenn.edu

Published 10 December 2021, *Sci. Adv.* **7**, eabj9141 (2021)
DOI: 10.1126/sciadv.abj9141

The PDF file includes:

Figs. S1 to S3
Legends for data files S1 to S3

Other Supplementary Material for this manuscript includes the following:

Data files S1 to S3

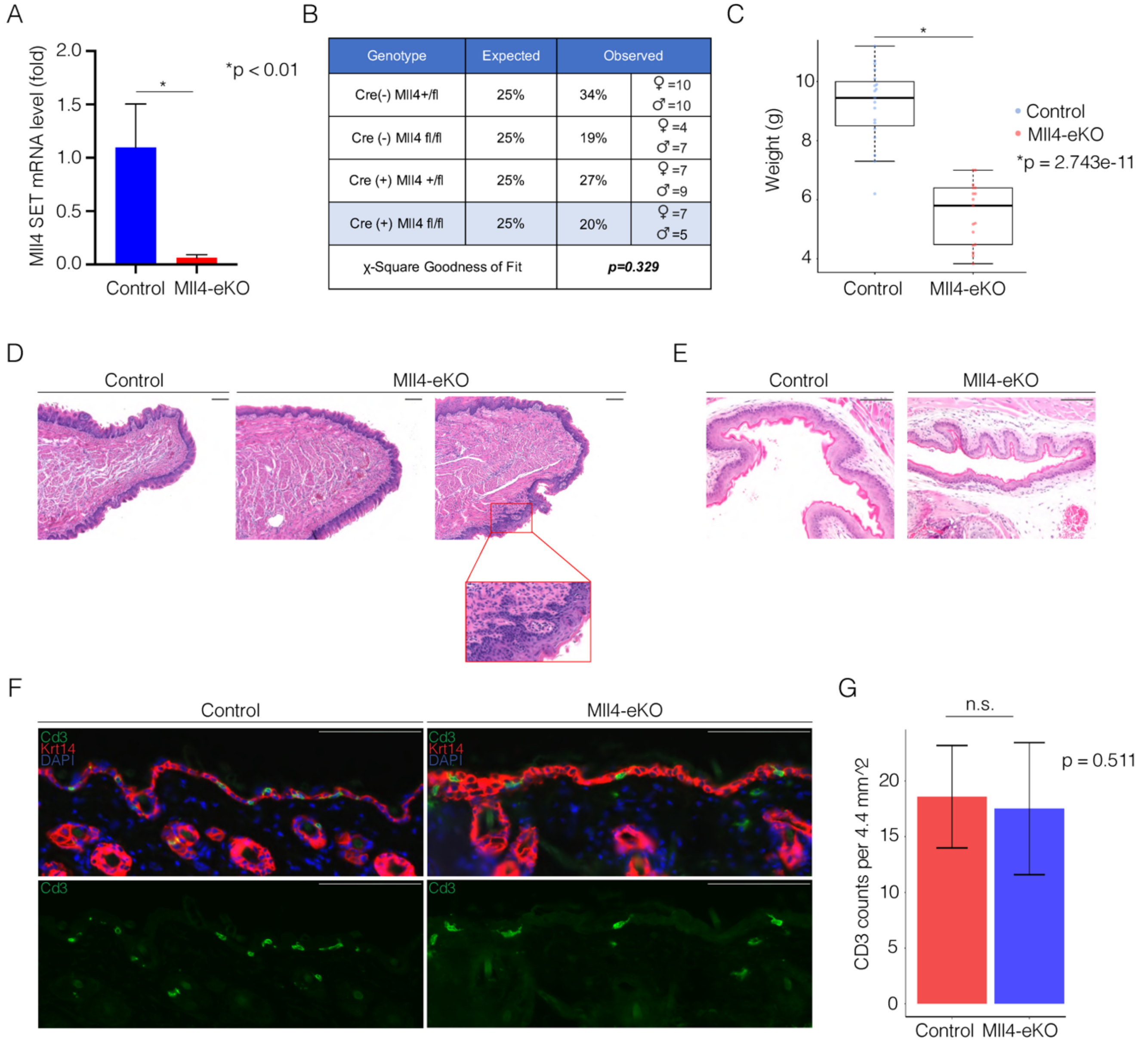

Fig. S1. (A) qRT-PCR of Mll4-SET expression in isolated bulk epidermis of 3-week-old Mll4-eKO and control mice (n=3-4 mice per genotype, p<0.01). (B) Expected and observed percentiles of mendelian ratio birth rates of Mll4-eKO mice (p=0.329). (C) Weights of 3-week-old Mll4-eKO (red) and control (blue) mice (n=17-19 mice per genotype, p=2.743e-11). (D) H&E histological staining of 3-week-old Mll4-eKO and control mice tongue (n=3 mice per genotype). (E) H&E histological staining of 3-week-old Mll4-eKO and control mice esophagus (n=3 mice per genotype). (F) IF staining of 3-week-old Mll4-eKO and control mice epidermis for Keratin-14 (red), CD3 (green), and DAPI (blue) (n=2 mice per genotype). (G) Quantification of total CD3 positive cells per 4.4mm^2 of images collected in F (p=0.511). Scale bar: 100 µM unless otherwise noted.

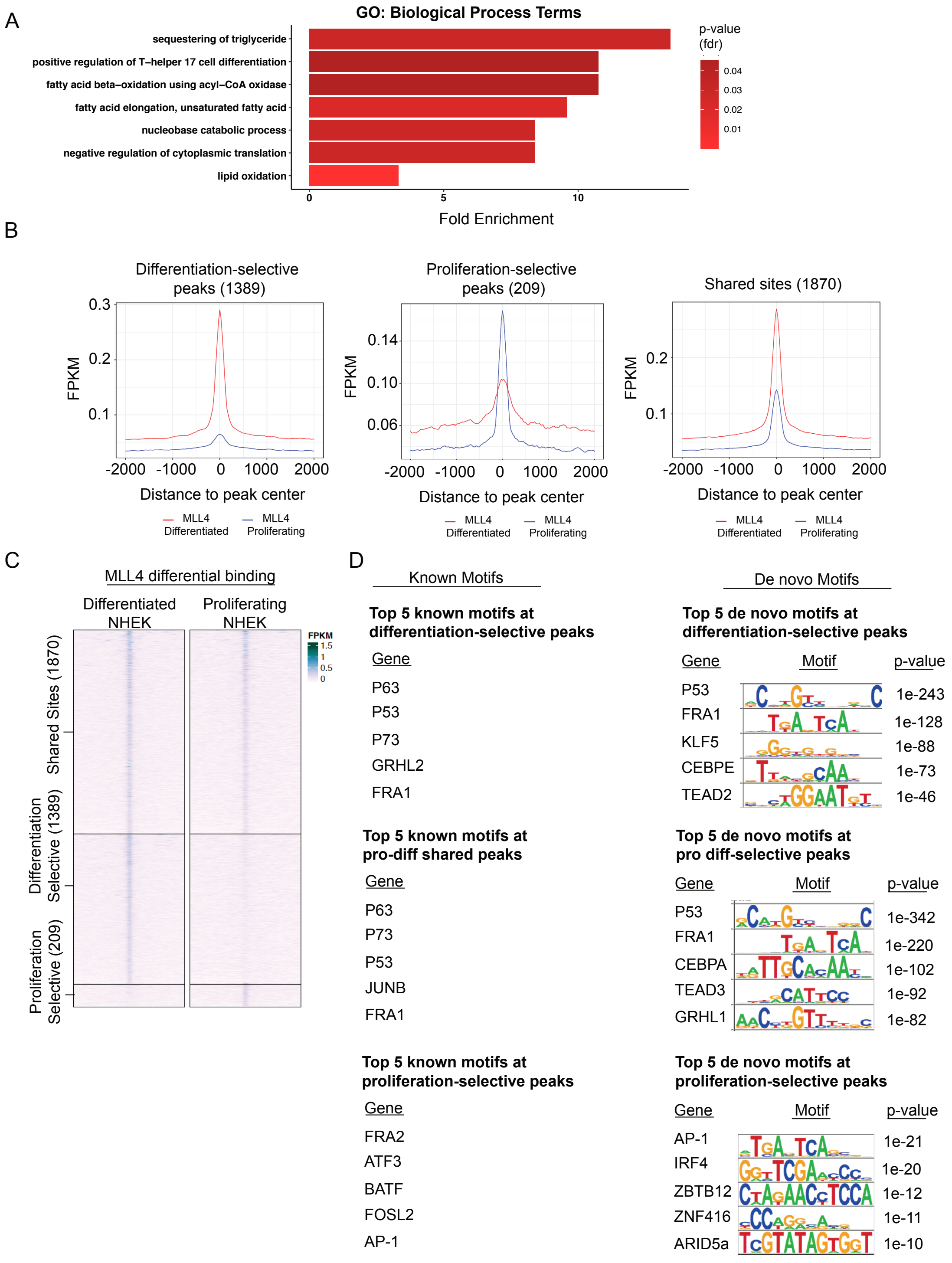

Fig. S2. (A) GO biological process analysis of Mll4-eKO differentially expressed upregulated genes (1687 genes). (B, C) Comparison of MLL4 binding intensities at differentiation-selective, proliferation selective, or shared sites in differentiated (red, n=3) or proliferating (blue, n=2) human keratinocytes by average profiles (B) or heatmap (C). (D) Top known (left) or *de novo* (right) motifs enriched at MLL4 binding sites at differentiation-selective, proliferation-selective, or shared sites.

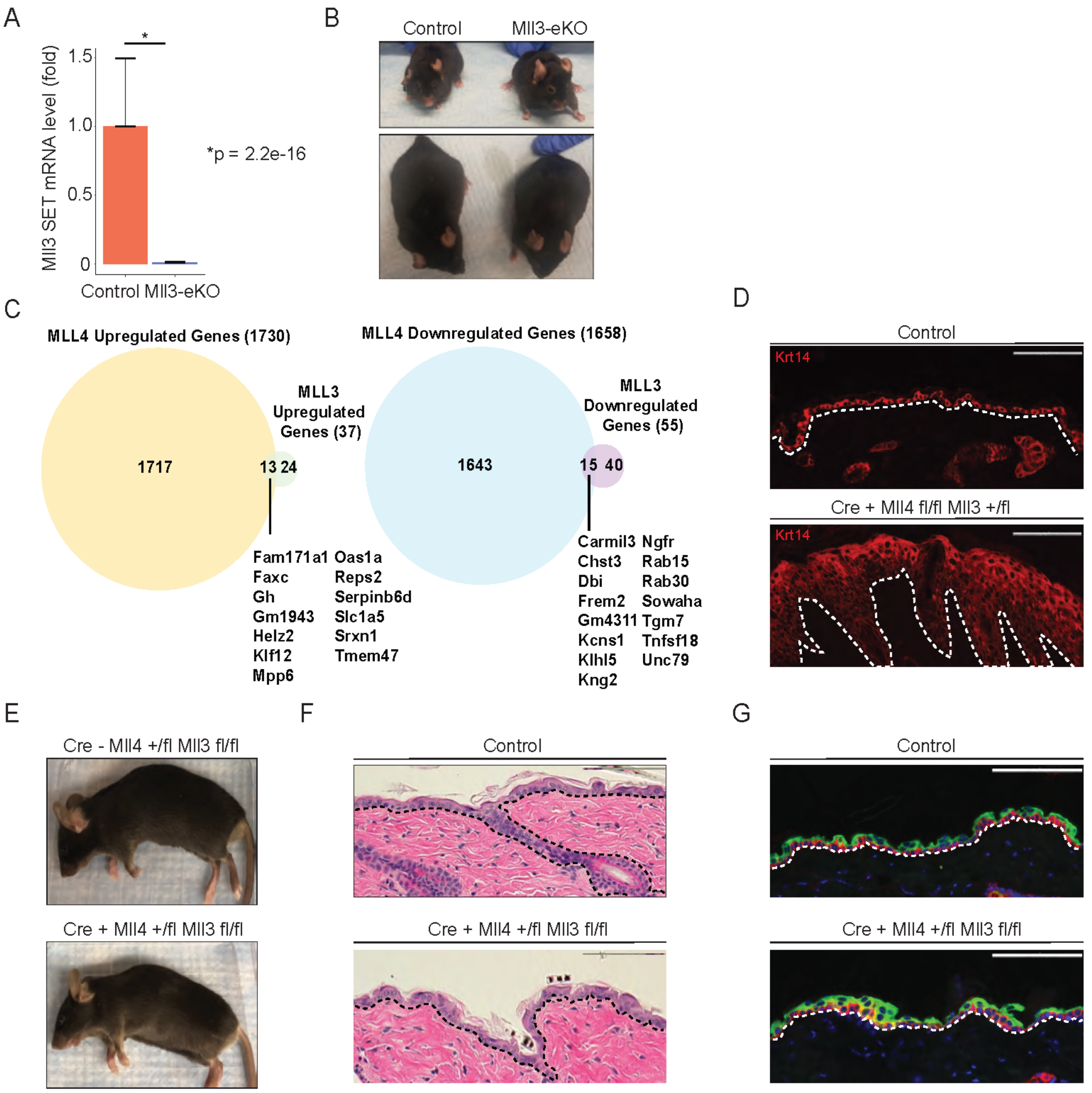

H

Genotype			Expected	Observed
Cre	Mll4	Mll3		
-	+/fl	+/fl	12.5%	12%
-	fl/fl	+/fl	12.5%	23%
-	+/fl	fl/fl	12.5%	20%
-	fl/fl	fl/fl	12.5%	15%
+	+/fl	+/fl	12.5%	3%
+	fl/fl	+/fl	12.5%	12%
+	+/fl	fl/fl	12.5%	15%
+	fl/fl	fl/fl	12.5%	0%

Fig. S3. A) qRT-PCR of Mll3-SET expression in isolated bulk epidermis of 3-week-old Mll3-eKO and control mice (n=3-4 mice per genotype. p<0.01)**. (B)** Representative 1–year-old Mll3-eKO and control mice. **(C)** Venn diagrams showing overlapping genes between Mll4-eKO and Mll3-eKO upregulated (left) or downregulated (right) genes. **(D)** IF staining of Krt14-Cre(+); *Mll4*$^{fl/fl}$; *Mll3*$^{+/fl}$ and control mice epidermis for Keratin-14 (red), and DAPI (blue) (n=3 mice per genotype)**. (E)** Representative adult Krt14-Cre(+); *Mll4*$^{fl/+}$; *Mll3*$^{fl/fl}$ and control mice**. (F, H)** H&E histological staining of Krt14-Cre(+); *Mll4*$^{fl/+}$; *Mll3*$^{fl/fl}$ and control epidermis (n=3 mice per genotype). **(G)** IF staining of Krt14-Cre(+); *Mll4*$^{fl/+}$; *Mll3*$^{fl/fl}$ and control mice epidermis for Keratin-14 (red), Keratin-10 (green), and DAPI (blue) (n=3 mice per genotype)**. (H)** Expected and observed percentiles for the offspring resultant from crosses between Krt14-Cre(+); *Mll4*$^{fl/+}$; *Mll3*$^{fl/+}$ and Krt14-Cre(-); *Mll4*$^{fl/fl}$; *Mll3*$^{fl/fl}$ mice (n=35 mice total). Scale bar: 100 µM unless otherwise noted.

Data S1. (separate file)
MLL4-eKO RNA-seq

Data S2. (separate file)
MLL4 ChIP-seq

Data S3. (separate file)
MLL3-eKO RNA-seq
